# The role of proteasomes in tumorigenesis

**DOI:** 10.1016/j.gendis.2023.06.037

**Published:** 2023-08-06

**Authors:** Xiangyi Zhou, Ruqing Xu, Yue Wu, Li Zhou, Tingxiu Xiang

**Affiliations:** a Department of Oncology, The First Affiliated Hospital of Chongqing Medical University, Chongqing 400016, China; b Chongqing Key Laboratory of Translational Research for Cancer Metastasis and Individualized Treatment, Chongqing University Cancer Hospital, Chongqing 400030, China

**Keywords:** Bortezomib, Cancer therapy, Immunoproteasome, Multiple myeloma, Proteasome 20S, Proteasome inhibitor, Thymoproteasom

## Abstract

Protein homeostasis is the basis of normal life activities, and the proteasome family plays an extremely important function in this process. The proteasome 20S is a concentric circle structure with two α rings and two β rings overlapped. The proteasome 20S can perform both ATP-dependent and non-ATP-dependent ubiquitination proteasome degradation by binding to various subunits (such as 19S, 11S, and 200 PA), which is performed by its active subunit β1, β2, and β5. The proteasome can degrade misfolded, excess proteins to maintain homeostasis. At the same time, it can be utilized by tumors to degrade over-proliferate and unwanted proteins to support their growth. Proteasomes can affect the development of tumors from several aspects including tumor signaling pathways such as NF-κB and p53, cell cycle, immune regulation, and drug resistance. Proteasome-encoding genes have been found to be overexpressed in a variety of tumors, providing a potential novel target for cancer therapy. In addition, proteasome inhibitors such as bortezomib, carfilzomib, and ixazomib have been put into clinical application as the first-line treatment of multiple myeloma. More and more studies have shown that it also has different therapeutic effects in other tumors such as hepatocellular carcinoma, non-small cell lung cancer, glioblastoma, and neuroblastoma. However, proteasome inhibitors are not much effective due to their tolerance and singleness in other tumors. Therefore, further studies on their mechanisms of action and drug interactions are needed to investigate their therapeutic potential.

## Introduction

Proteasome 20S is the major proteolytic enzyme in eukaryotes, which plays an important role in the regulation of intracellular protein stability. It is a hollow barrel structure composed of 28 αβ subunits,^1^^,^^2^ which is proteolytically active and plays the primary function of protein degradation.^3^ 20S particle can bind to PA700 particle to play the role of ubiquitinating ATP-dependent protein degradation. It can also bind to PA28 particle to play an ATP-independent role in protein degradation. The ubiquitin proteasome system is involved in maintaining the stability of several proteins implicating in the regulation of cell cycle and division, DNA repair and transcription, the immune response and inflammation, antigen processing, differentiation, and cell development and apoptosis,^4^^,^^5^ especially in cancer cells.^6^ Generally speaking, normal cells remove excess and unfolded or misfolded proteins by protease-mediated hydrolysis, thus maintaining intracellular proteostasis.^7^ In the development and progression of cancer, abnormal protein degradation caused by proteases is one of the significant causes, depending on the different tumor signaling pathways, and proteasomes also play various roles in different pathways.^8^, ^9^, ^10^, ^11^ It is now known that proteasomes are also involved in processes related to tumorigenesis as well as affecting penetration into non-growing cells, especially cancerous ones. In addition, immunoproteases and thymoproteasomes play important roles in the selection of CD8^+^ T cells, which provides a new idea for tumor immunotherapy.^12^^,^^13^ Today, scientists have confirmed the links between abnormalities of proteasome function and various diseases such as multiple myeloma (MM) and glioblastoma. Therefore, targeting proteasomes or regulating the function of intracellular protein through proteasomes may be an effective way of tumor therapy.^14^, ^15^, ^16^ So far, bortezomib has been approved by the FDA as a useful drug for the treatment of MM, but its efficacy in other tumors is still in the clinical research stage. In this review, we will focus on the source, structure, and function of the proteasome and emphasize its progress in tumor research in recent years, as well as its application and prospects in clinical practice.

## How are proteasomes discovered?

Early biologists thought that proteins were stable and hardly suffered damage from external factors. Until the 1930s, Rudolf et al confirmed that protein stability was due to a balance between synthesis and degradation.^17^ In the 1950s, the discovery of lysosome dynamic balance established a new understanding of protein decomposition.^18^^,^^19^ By 1953, Simpson suggested that there may be two mechanisms for protein degradation: “one hydrolytic, the other energy-requiring".^20^ Twenty years later, Etlinger and Goldberg discovered a new soluble ATP-dependent protein hydrolysate, which is independent of the lysosome system.^21^ However, the roles of ATP-dependent protein degradation and lysosomal decomposition were not clear at that time.

In 1983, Wilk and Orlowski first discovered a large “polycatalytic protease” complex containing chymotrypsin and proteinase-like activity in the pituitary gland. This complex did not exist in lysosomes, but in the cytoplasm.^22^^,^^23^ Later, a number of names for this complex have appeared in the literature, including polycatalytic protease, polycatalytic endopeptidase complex, ATP-stimulated alkaline protease, potentially alkaline multifunctional protease, “macropain”, but we prefer to call it “proteasomes".^24^, ^25^, ^26^, ^27^, ^28^, ^29^

## What is the structure of proteasomes?

The proteolytic activity in eukaryotic cells is a 700-kDa enzyme complex that is present in the nucleus and cytoplasm of almost all mammalian cells, as well as in yeast and Drosophila.^30^

Generally speaking, a complete proteasome holoenzyme consists of one core particle with other regulate particles. The core particle is also called proteasome 20S, a hollow tubular structure composed of four heptamer rings, which include two outer rings composed of 7α subunits and two inner rings composed of 7β subunits,^31^^,^^32^ they overlap to form the structure of concentric circles. The X-ray analysis has confirmed that the proteasome 20S particle is a barrel-shaped cylinder composed of a penetrating central channel, three large internal chambers, and a cavity where the misfolded protein can be controlled to enter.^33^ The α rings have no activity, they only play a gating role for proteasome 20S. In fact, the orifices formed by the α ring at the top are very narrow so that they can restrict protein to get inside it.^34^ The β rings are the main catalytic subunit, and it is formed by a circle of β1-7. The subunits of β3, β4, β6, and β7 are discordant parts of consisting complete β ring structure, and without their participation, the rate of protein degradation will be reduced. However, the main active subunits β1, β2, and β5 subunits hold three distinct proteolytic activities consisting of caspase-like activity, trypsin-like activity, and chymotrypsin-like activity.^35^^,^^36^

The subunits of the 19S regulate particles functionally control the translocation of ubiquitinated substrates, they consist of a base with ATPase activity and a lid without ATPase activity.^37^^,^^38^ The base subunits participate in substrate binding, unfolding, and translocation, while the lid subunits serve as ubiquitin receptors and are necessary for the binding of ubiquitylated substrates.^39^^,^^40^ The shape of 11s is just like a hat that stimulates the degradation of peptides by opening a channel of the exterior of the proteasome. Unlike the 19S regulators, 11S regulators do not deliver substrate but instead promote the product peptides exit from the interior of the 20S proteasome.^41^^,^^42^

**Figure 1:**
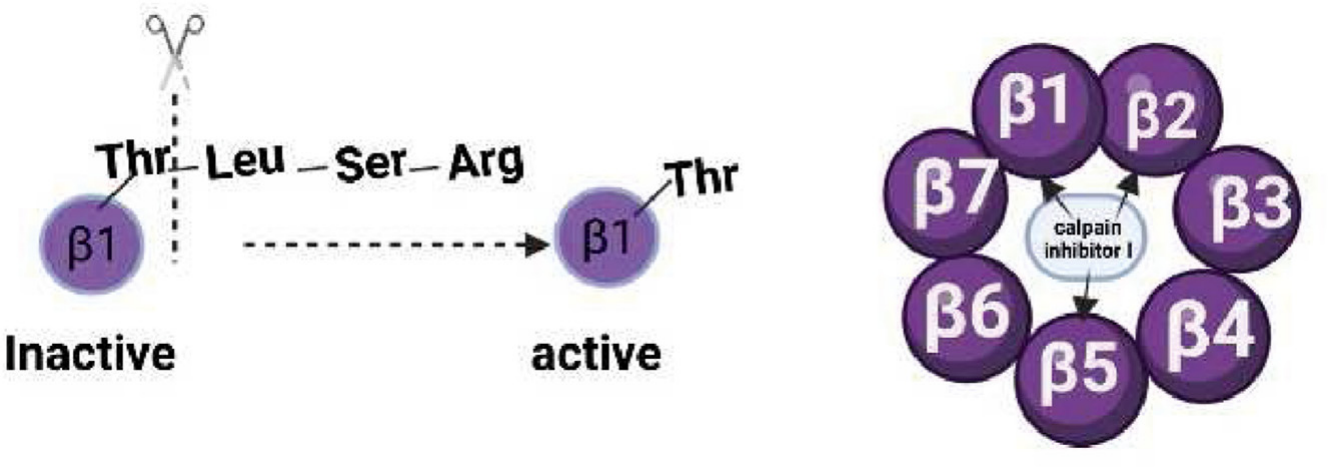
The self-degradation of threonine-dependent propeptides exposed the terminal threonine, and the β subunit changed from inactive to active. Calpain inhibitors I can bind to several amino acids at the ends of β1, β2, and β5.

Nevertheless, how do these three subunits perform the role of protein degradation? Emerging evidence indicated that several enzymes have no activity when they are synthesized in the cell, which we called propeptides. In some situations, the propeptides are interrupted by one or more special peptide bonds, leading to certain conformational changes, which we termed active enzymes. The terminal threonine residue of the β subunit is conserved in most eukaryotes, but during the process of biosynthesis, the self-degradation of threonine-dependent propeptides was triggered, and then the terminal threonine is exposed.^43^ This is the assembly of active 20S proteasome. In 1997, Michael et al^2^ extracted proteasome 20S crystals from yeast, and they found that calpain inhibitors I with acetyl-Leu-Leu-norleucinal sites can covalently bind to Thr-1, the side chains of calpain inhibitors I project to several amino acids at the ends of β1, β2, and β5 to form pocket-like structures.^33^ They exactly are the active sites of the proteasome after the propeptide modification. Interestingly, through binding to the inhibitors, the activities of proteasome 20S are greatly blocked^44^ (Fig. 1).

The proteasome 20S is just like a garbage can, which can bind to different subunits to perform different catalytic functions. It is involved in numerous biological processes, including the removal of aberrant proteins, stress response, cell-cycle control, cell differentiation, and cellular immune responses.^4^, ^5^, ^6^

## How do proteasomes perform their function?

The proteasome 20S simply degrades the unfolded protein in an energy-independent manner, and it must be bound to the 19S subunit to complete its function.^30^^,^^45^ The regulation particle of 19S consists of a cap-like structure composed of a “cover” and a “base”, which are embedded at both ends of the 20S catalytic core to form a “dumbbell-like” symmetrical structure.^34^^,^^46^ When 20S binds to the 19S subunit, also known as PA700, it selectively degrades ubiquitinated proteins by an ATP-dependent process,^47^ generally called the proteasome 26S. This is currently the most extensively studied proteasome system, and most proteins in cells are degraded by proteasome 26S system.^48^ Just as we mentioned before, the opening of the α ring is just like a “door” that resists normal folded proteins entering inside,^1^^,^^49^ while the bottom of 19S is just like a key. When it binds to the top of the α ring, it can open the door and promote the protein into the cavity.^50^, ^51^, ^52^ The substrate carrying ubiquitin chain is recognized by the 19S cap structure, then enters the 20S active center to be degraded, and then is released from the other end of the proteasome.^53^

Another activator, PA28, also known as 11S regulator, can associate with the proteasome 20S in the absence of ATP.^54^ Electron microscopy demonstrates that PA28 is a ring-shaped particle, and like PA700, caps the 20S proteasome at both or either end.^54^ At the same time, it can also combine with 19S to form a “mixed proteasome”. PA28 can promote the hydrolysis of short peptides, this process is independent of ATP, and the hydrolyzed substrate does not need to be combined with ubiquitin. Although PA28-20S is not as extensive as 26S in degrading protein species, it is directly involved in the operation of the adaptive immune system.^55^

**Figure 2:**
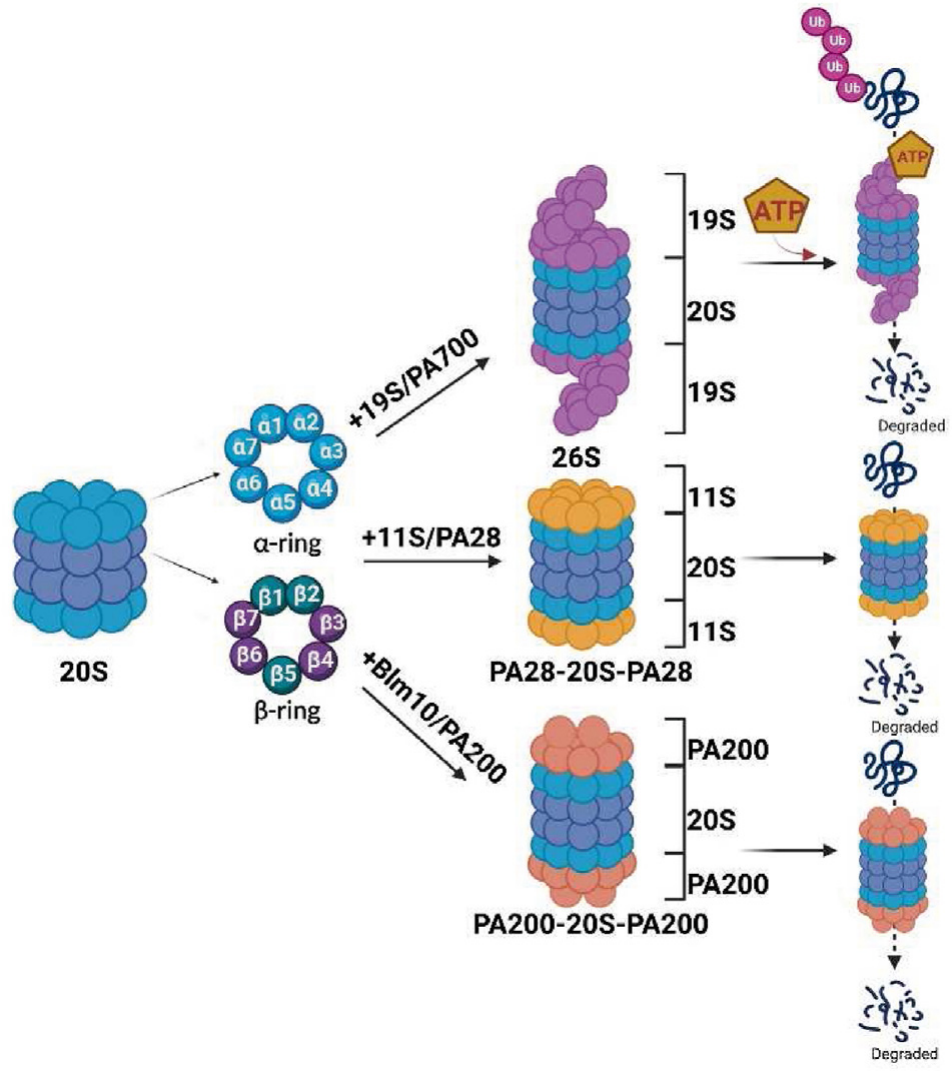
The proteasome 20S is a concentric circle structure with two α rings and two β rings overlapping. By binding to various subunits, such as 19S, 11S, and 200 PA, it can form the proteasome 26S, PA28-20S-PA28, and PA200-20S-PA200, and execute the function of ATP-dependent and non-ATP-dependent ubiquitination proteasome degradation, which is performed by its active subunit β1, β2, and β5.

The third proteasome activator is PA200, also known as Blm10 in yeast. Similar to the two above, it binds to the end of 20S particle and acts as a key to open the “door” of the α-ring. It is mainly involved in the degradation of unstructured proteins, such as histones. The substrate does not need ubiquitin labeling in the process of degradation with PA200. However, recent studies have also found that PA200 plays an important role in maintaining histone code stability and delaying aging^56^ (Fig. 2).

## Proteasomes and tumor

Proteasome is the key complex of the ubiquitin-proteasome system, which is responsible for the degradation of redundant and misfolded proteins. It has been proved to be critical in the regulation of cell growth and oncogenic transformation.^57^^,^^58^

## The role of proteasomes in tumor progression

The progression of tumors involves cell cycle regulation, cell growth, apoptosis, *etc*. Proteasome inhibitors, as the first-line clinical treatment of MM, also affect the development of MM through multiple aspects. It seems that it can alter cell interactions and cytokine secretion in the tumor microenvironment to inhibit tumor cell growth and associated angiogenesis, induce apoptosis, and appear to overcome drug resistance.^59^^,^^60^ Next, we will introduce how proteasomes play a role in tumorigenesis and highlight the mechanism of proteasomes in MM.

## Proteasomes acted on tumor signaling pathway

One of the key tumor signaling pathways regulated by proteasomes is the NF-κB pathway.^61^ NF-κB is a transcription factor that plays a critical role in the regulation of immune responses, inflammation, and cell survival.^62^ Dysregulation of NF-κB has been implicated in the development and progression of many types of cancer.^63^ In the classical pathway, activation of the IκB kinase complex (IκB kinase β/α/γ), leads to the phosphorylation of IκB, an inhibitor of NF-κB, which makes IκBα ubiquitinated and recognized by proteasomes.^64^, ^65^, ^66^, ^67^ When the proteasomes degrade the inhibitory protein IκB, NF-κB is transferred to the nucleus to promote gene transcription and cell growth.^68^ In other words, proteasome inhibitors can block the degradation of IκB, leading to the inhibition of NF-κB activity and the induction of apoptosis in cancer cells.^69^

The other tumor signaling pathway regulated by proteasomes is the Wnt/β-catenin pathway. β-catenin is a transcriptional co-activator that plays a critical role in the regulation of cell proliferation and differentiation.^10^ Dysregulation of β-catenin has been implicated in the development and progression of many types of cancer.^11^ Without the presence of Wnt, free β-catenin is bounden with adenomatous polyposis coligene (APC) product, Axin, glycogen synthase kinase 3β (GSK3β) and CK1, which is also called β-catenin destruction complex.^70^^,^^71^ Subsequent phosphorylation of β-catenin by GSK3β and CK1 drives β-catenin ubiquitination and is responsible for promoting degradation via the proteasome pathway.^72^

Activation of Wnt/β-catenin signaling has been reported to contribute to cancer development and progression.^73^^,^^74^ Thus, suppressing the excessive activation of the Wnt/β-catenin signaling pathway must be a key therapeutic method.^75^^,^^76^ In previous study, some natural products such as epigallocatechin-3-gallate have been found to inhibit the Wnt/β-catenin signaling by promoting the phosphorylation and ubiquitination of β-catenin, leading to the degradation of β-catenin in the tumor cells.^77^, ^78^, ^79^, ^80^

**Figure 3:**
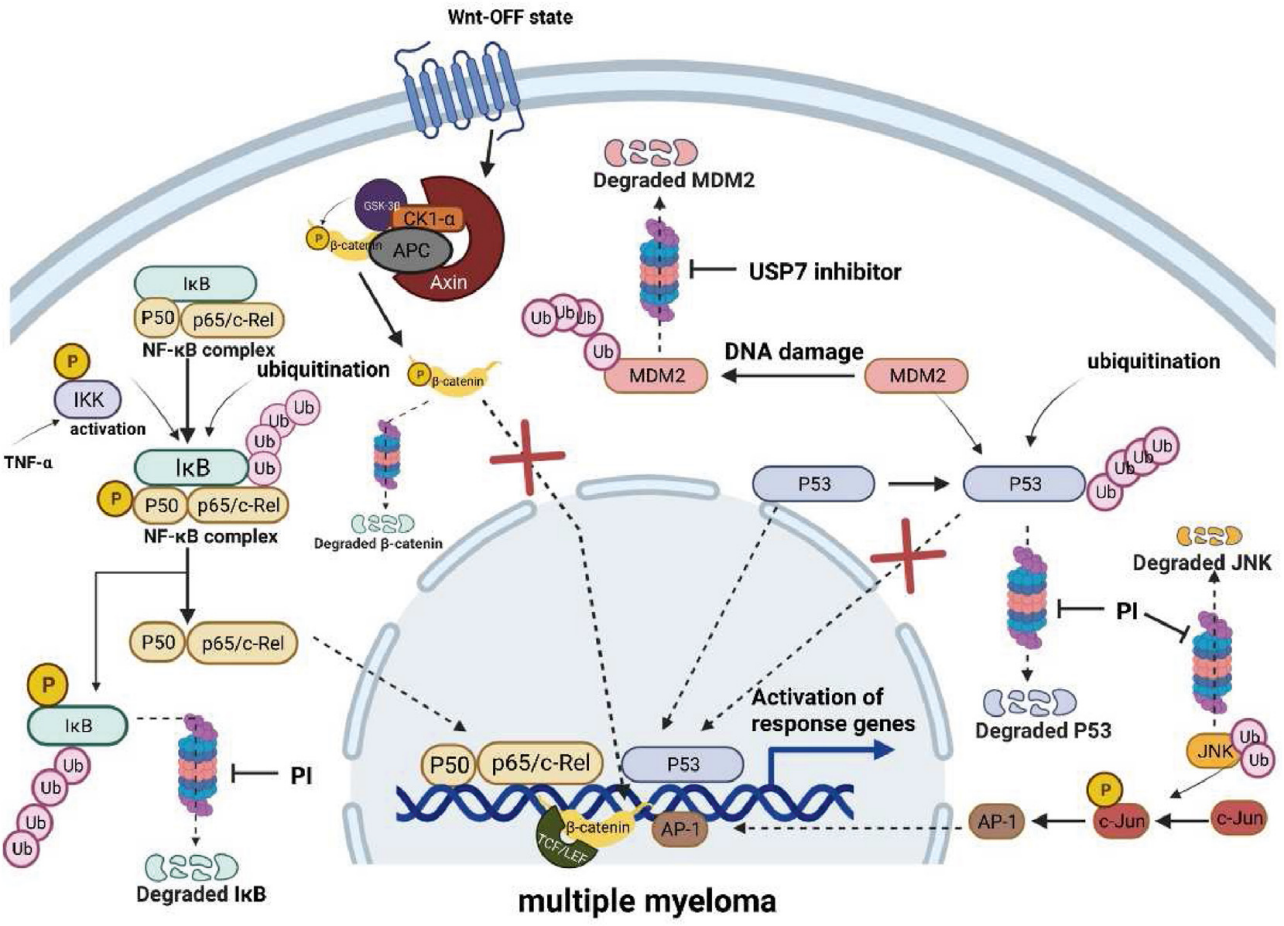
The role of proteasomes in tumor signaling pathways. Free β-catenin is linked to adenomatous polyposis coligene products (APCs), Axin, glycogen synthase kinase 3β (GSK3β) and CK1 in the absence of Wnt. Subsequent phosphorylation of β-catenin by GSK3β and CK1 drives β-catenin ubiquitination and is responsible for promoting degradation via the proteasome pathway. Activation of the IκB kinase complex leads to phosphorylation of IκBα, which leads to IκBα ubiquitination and degradation by proteasomes. Then NF-κB is transferred to the nucleus to promote gene transcription and cell growth. Proteasome inhibitors can inhibit the ubiquitin-protease system and reduce the degradation of IκB. Murine double minute 2 (MDM2) can reduce p53 transportation to the cytoplasm for ubiquitination degradation by binding to it, thus affecting the transcriptional activity of p53. When DNA is damaged, MDM2 can be degraded by self-ubiquitination, allowing the accumulation of p53. Ubiquitin-specific protease 7 (USP7) inhibitors can inhibit the ubiquitin-protease system and promote the degradation of MDM2. Proteasome inhibitors increased the level of JNK with the consequent increase in c-Jun phosphorylation. These events increase AP-1 activity, which can induce the expression of downstream genes and trigger apoptosis in tumor cells.

The p53 tumor suppressor protein is a critical regulator of cell cycle progression, apoptosis, and senescence.^81^ As a tumor suppressor gene, p53 mutation is very common in tumorigenesis. The activity of p53 is strictly regulated by changing homeostasis levels and post-translational modification. Proteasome inhibition gives rise to increased p53 expression and stability, thereby promoting the programmed death process.^82^ Murine double minute 2 (MDM2) is considered an effective inhibitor of p53.^83^, ^84^, ^85^, ^86^ Through binding to p53, it can reduce its transportation to the cytoplasm for ubiquitination degradation, thus affecting the transcriptional activity, stability, and subcellular localization of p53.^87^^,^^88^ When DNA is damaged, MDM2 can be degraded by self-ubiquitination, allowing the accumulation of p53.^89^^,^^90^ In view of MDM2 playing a key role in regulating the growth, proliferation, and cell cycle progression of cancer cells, drugs targeting the ubiquitin-protease system of p53 and MDM2, such as ubiquitin-specific protease 7 (USP7) inhibitors and SP141 have a good prospect of therapy.^8^^,^^9^^,^^91^ The mechanism of it is promoting the degradation of MDM2, thus activating the p53 signaling pathway and causing cell cycle arrest and apoptosis^92^ (Fig. 3).

An important role in the control of the apoptotic mechanism is exerted by the c-Jun/JNK/AP-1 pathway. Proteasome inhibition will lead to the activation of JNK, which is an important activator of phosphorylated c-Jun. When it is activated, with c-Fos, it forms a dimer AP1; a transcription factor involved in the regulation of several cell processes,^93^^,^^94^ which can induce the expression of downstream genes such as Fas-L and trigger apoptosis in tumor cells.^95^

In addition to the several signaling pathways we mentioned above, proteasomes also regulate other tumor signaling pathways, such as the PI3K/Akt/mTOR pathway, the Notch pathway, and the Hedgehog pathway.^96^, ^97^, ^98^ There are also close links between different signaling pathways, for example, β-catenin overexpression protects p53 protein from MDM2-mediated proteasome degradation, thereby stabilizing p53 protein and increasing its transcriptional activity.^99^^,^^100^ However, the activation of p53 down-regulates β-catenin through GSK3β phosphorylation mediated proteasome degradation.^101^^,^^102^ Proteasome plays a critical role in the regulation of tumor signaling pathways and dysregulation of proteasome activity has been implicated in the development and progression of many types of cancer.^16^^,^^103^, ^104^, ^105^ Generally speaking, proteasomes also play different roles in tumor cells according to different tumor signaling pathways. For example, signaling pathways that activate tumor proliferation and development require overexpressed proteasomes to down-regulate their expression, whereas, for the down-regulation of signaling pathway factors that deter tumor growth, proteasome inhibitors are needed to reduce their degradation.

## Proteasomes acted on cell cycle

The cell cycle is a necessary process for cell proliferation. According to their different modes of function, cell cycle regulation can be divided into positive regulators and negative regulators. If negative regulators such as CKI are not degraded in time, cells will be arrested in a certain cycle, resulting in cell death and misdifferentiation. Positive regulators, such as cyclin and CDK, can generally promote the process of cell division. If they are not degraded in time, abnormal cell differentiation will lead to tumor.^106^, ^107^, ^108^, ^109^ Given that ubiquitin-proteasome pathway-mediated cyclin hydrolysis plays an important role in this process, the degradation of cycle-regulated proteins is abnormal in many cancers. Furthermore, p21 family members (p21, p27, and p57) could inhibit CDK complex activity and affect progression of the cell cycle. Low levels of p21 and p27, brought about by proteasome hyperactivity, represent a negative prognostic factor in several types of cancers. As a result, inhibiting action by the proteasome leads to up-regulation of these proteins, which may lead to apoptosis in tumoral cells *in vitro*.^110^, ^111^, ^112^ Given that the cyclin-D1-CDK4 complex is required for the G1-S phase and cyclin B1-CDK1 complex is required for mitosis,^113^, ^114^, ^115^ proteasome inhibitor prevents the proliferation of hepatocellular carcinoma cells during these phases by inhibiting cyclins A1, B1, and D1, as well as CDKs1 and 4.^114^, ^115^, ^116^ It can also prevent cell cycle progression via the reduction of hyperphosphorylated E2F1 and cyclin D1, which is associated with an up-regulation of the cell cycle inhibitors p27^Kip1^ and p21^waf1/cip1^.^117^ In addition, studies have shown that CDK15 may be the downstream target of PA28, the down-regulation of PA28 can effectively inhibit the invasion and metastasis of breast cancer, and save the expression of CDK15 protein, but the silencing of CDK15 can, in turn, improve the inhibition of PA28. But the exact molecular mechanism between them needs further study.^118^

## Immunoproteasome and drug resistance

**Figure 4:**
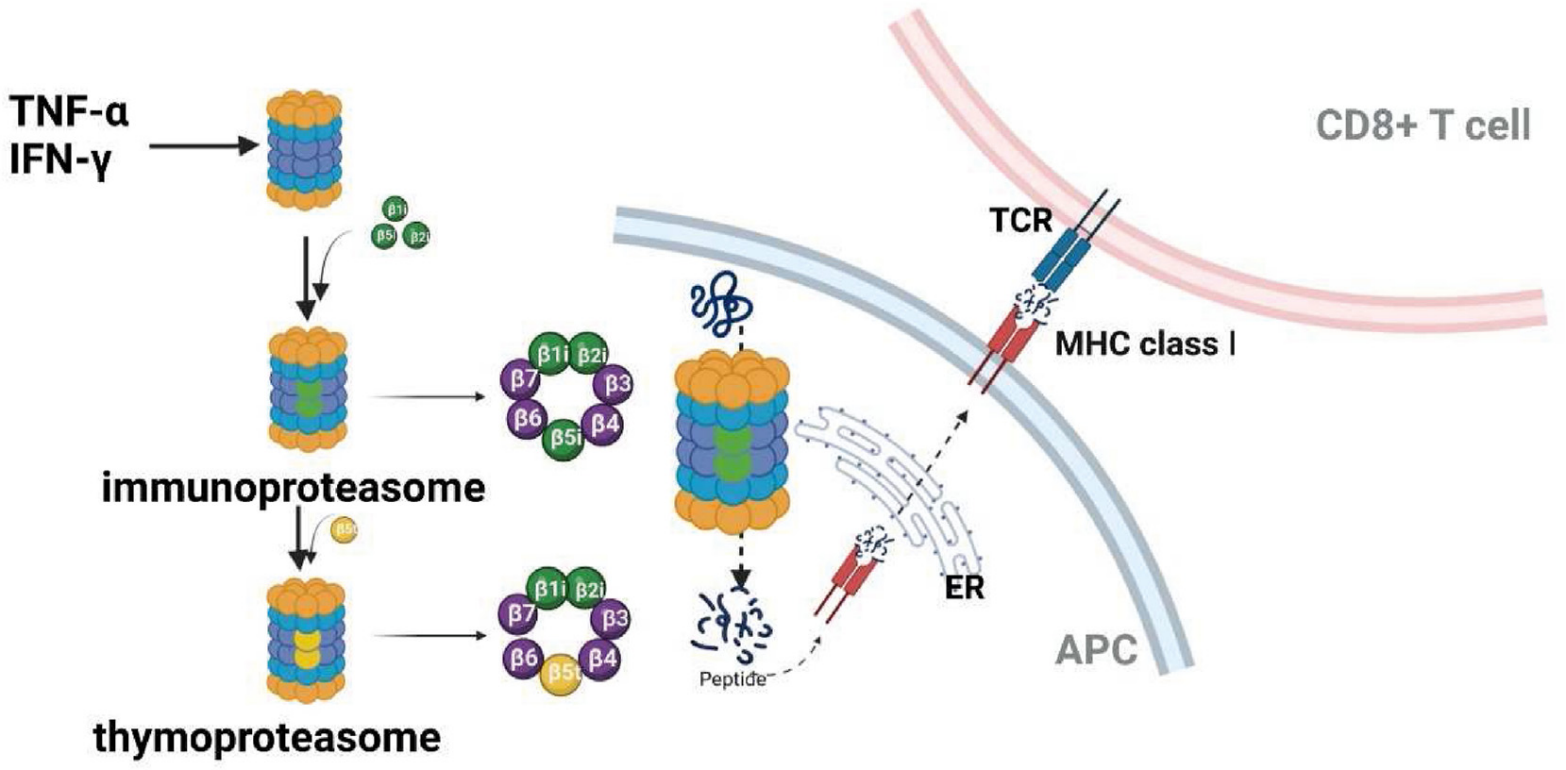
Tumor necrosis factor (TNF)-α and interferon (IFN)-β can induce the production of immunoproteasome β1i, β2i, and β5i and thymoproteasome β1i, β2i, and β5t, which contribute to the degradation of the invasive pathogens, following by changing those pathogens into peptide segments with appropriate size for major histocompatibility complex (MHC) class I binding in the endoplasmic reticulum (ER), deliver them to the surface of antigen-presenting cells (APCs), and then pass the signal to the CD8^+^ T cell. TCR, T cell receptor.

When the immune response occurs, interferon-γ and tumor necrosis factor-α can induce the expression of PA28 and special β subunits (β1i, β2i, and β5i),^119^ forming a special structure, we called it “immunoproteasome".^120^ It can degrade the invading pathogens into peptide segments of appropriate size for binding to major histocompatibility complex (MHC) class I in the endoplasmic reticulum (ER), deliver them to the surface of antigen-presenting cells,^121^ and then pass the signal to the CD8^+^ T cell^13^^,^^122^^,^^123^ (Fig. 4). Thus, the immunoproteasome β1i, β2i, and β5i subunits play an important role in preventing virus infection. However, its deficiency may also protect cancer cells from immune attack,^121^^,^^124^, ^125^, ^126^, ^127^, ^128^, ^129^, ^130^ immune proteasome can be used to replace constitutive proteasome (β1, β2, and β5) to exercise the role of protein degradation. Therefore, large numbers of immune proteasomes may promote tumor growth and lead to resistance to proteasome inhibitors.^131^, ^132^, ^133^, ^134^

The seemingly contradictory role of immune proteasomes provides a new insight for tumor targeting drugs, but it also requires us to have a good understanding of the mechanism according to different types of cancer and the functions of proteasomes.

For example, LMP7, one of the functional subunits of immunoproteasome β5i, encoded by PMSB8, significantly reduced the tumor burden in colon cancer mice and blocked tumor initiation and progression.^133^ Interestingly, as a β5i inhibitor, PR-924 also has a significant effect on the growth inhibition and inducing apoptosis of MM *in vivo* and *in vitro*. In addition, PR-924 treatment can prolong the survival time of tumor-bearing mice.^135^ In the early stage of non-small cell lung cancer, low expression of immunoproteasome subunits can enable tumor cells to evade immune surveillance, which not only causes tumor cell recurrence and metastasis but also reduces disease-free survival. Induction of immunoproteasome by IFN-γ or 5-aza-2′-deoxycytidine can overcome the immune escape mechanism of mesenchymal cells by restoring the function of HLA class I-bound peptides.^136^

## Thymoproteasome: An essential part of CD8^+^ T cell optimal generation

The thymus is an essential part of T cell development, from which we detected a special proteasome termed thymoproteasome in cortical thymic epithelial cells.^12^^,^^137^, ^138^, ^139^ Thymoproteasome is characterized by β5t,^12^^,^^140^^,^^141^ which generates a unique spectrum of MHC class I-associated peptides and plays a critical role in thymic positive selection of CD8^+^ T cells.^12^ Though thymoproteasomes have similar structures to immunoproteasomes, which consist of β1i, β2i, and β5t subunits (Fig. 4), the hydrophilic residues in β5t have lower chymotrypsin-like activity compared with β5i, which exhibits a unique substrate specificity in endopeptidase proteolysis.^12^^,^^140^^,^^142^ Through the analysis of β5t-deficient mice, we will discover a significant decrease of CD8^+^ T cells whereas no obvious change was seen in CD4^+^ T cells, NKT cells, B cell DCs, and other cells compared with control group.^12^^,^^143^ Because proteasomes are responsible for the production of relative peptides and are presented by CD8^+^ T cells, it makes sense that thymoproteasome is able to display a unique set of peptides associated with cell-surface MHC class I molecules and positive recognized by CD8^+^ T cells.^144^, ^145^, ^146^, ^147^, ^148^ However, the mechanism for thymoproteasome-dependent CD8^+^ T cell production in the thymus remains unclear. There have been two main hypotheses that revealed how CD8^+^ T cells selected peptides degraded by thymoproteasome specifically. One of the hypotheses is “self-peptide",^149^^,^^150^ which showed that thymoproteasome-dependent-self-peptide may create a difference between cortical thymic epithelial cells and other antigen-presenting cells. This difference provides a chance for positively selected CD8^+^ T cells to escape from a negative selection of other cells.^147^^,^^151^ The second hypothesis is “low affinity motif”; consistent with this view, thymoproteasome-dependent MHC class I-associated peptides have low–affinity interaction with T cell receptors, which promotes the positive selection of CD8^+^ T cells.^141^^,^^148^^,^^152^^,^^153^

As β5t is expressed in most cases of type B but not type A, thymoproteasomes are also a detective target useful in differentiating type A from type B thymoma. Morphologically, type A and type B thymomas exhibit differentiation into mTECs and cortical thymic epithelial cells, respectively. Therefore, β5t retains its normal physiologic expression pattern in the context of thymoma and is a reliable marker for detecting neoplastic epithelial cells differentiating into cortical thymic epithelial cells.^154^, ^155^, ^156^

In terms of such advantages, research on the immunoproteasomes and thymoproteasomes may contribute to improving the efficacy of immune checkpoint inhibitors as well as therapies for other immune disease. Nevertheless, the development of an inhibitor specific to thymoproteasomes has also been attempted but has not yet succeeded. The development of inhibitors specific to immunoproteasomes would provide a promising tool for treating cancer and immunological diseases in the future.

## Regulation of tumor by proteasome-encoded genes

At the genetic level, many proteasome subunit genes are closely related to tumor progression. A variety of genes encoding the proteasome subunit have been found to be up-regulated in various tumors.^157^, ^158^, ^159^, ^160^ In 2007, Deng et al analyzed the difference in proteasome genes between breast cancer tissues and normal tissues by RT-PCR and found that several 26S particle subunits coding genes were up-regulated, including PSMB5, PSMD1, PSMD2, PSMD8, and PSMD11.^161^ Hepatitis B virus is one of the most common risk factors for liver cancer. Interestingly, it has been reported that proteasome is a potential cellular target of HBx.^162^ Many previous studies have shown that HBx interacts with proteasome subunits (including PSMA1, PSMA7, PSMB7, PSMC1, and PSMC3^163^) which influence hepatitis B virus replication through the proteasome-dependent pathway.^164^ PMSF7 encodes the proteasome α subunit and its mRNA level was found to be significantly different between colorectal and normal tissues by RT-PCR. PMSF7 expression was elevated in colorectal cancer as well as metastasis and negatively correlated with survival.^165^ The protein subunit encoded by PSMD2 is a part of the 19S particle, which is essential for the assembly of both the 19S and 20S proteasomes.^166^^,^^167^ Studies have demonstrated that PSMD2 is significantly up-regulated in breast cancer and is associated with poor prognosis. Knockdown of PSMD2 in breast cancer cells can effectively inhibit cell proliferation and arrest cell cycle at G0/G1, which is caused by the up-regulation of p21 and p27. In terms of mechanism, PSMD2 interacts with p21 and p27 and mediates ubiquitin-proteasome degradation in coordination with USP14.^168^

Tumor drug resistance is also a key factor in cancer treatment failure. Studies have found that in microarray samples of 1592 breast cancer patients, PSMB7 (gene encoded β5) has been found to be overexpressed in breast cancer cells and associated with poor prognosis. Interestingly, by comparing the expression profiles of adriamycin resistance in four different pairs of human tumor cell lines, PSMB7 overexpression could be found in the drug-resistant cell lines, and the resistance of the cells was weakened after PSMB7 was silenced by RNA interference.^169^ This provides a new idea for the mechanism of cell drug resistance. Perhaps we can use proteasome inhibitors to target cell drug resistance.

## Proteasome-targeted tumor therapy

Proteasome inhibitors, on the one hand, can interfere with the protein metabolism of tumor cells, making the tumor overburdened; second, they can relieve the degradation of tumor suppressor factors, thus playing a role in tumor treatment. In addition, proteasome inhibitors are more sensitive to tumor cells than normal cells, which can prevent tumor cell proliferation, selectively induce tumor cell apoptosis, and increase the sensitivity to radiotherapy and chemotherapy.

Excessive proliferation of tumor cells requires proteasome to maintain homeostasis to eliminate the accumulation of misfolded proteins. For example, MM originates from the hyperproliferation of bone marrow plasma cells, which makes malignant plasma cells secrete more immunoglobulins than normal plasma cells. Immunoglobulin is a macromolecule synthesized and folded in the ER.^170^ The production of high immunoglobulins makes MM cells heavily dependent on proteasomes to maintain ER homeostasis, so they are more sensitive to proteasome inhibition.^171^ So far, some proteasome inhibitors have been approved by the FDA for the treatment of tumors. It is widely known that bortezomib, carfilzomib, and ixazomib, are used in the treatment of MM and mantle cell lymphoma (Table 1).

**Table 1:** Proteasome inhibitors for targeted tumor therapy.

Proteasome inhibitor	Substitute name(s)	Targeted tumors or diseases	Advantages	Disadvantages	References
Bortezomib	PS-341, MG-341, Velcade	New diagnosis multiple myeloma, pancreatic cancer, hepatocellular carcinoma, non-small cell lung cancer, breast cancer, renal cancer, head and neck squamous cell carcinomas, ovarian cancer	Wide range of indications; various treatment schemes	High drug resistance rate; high incidence of peripheral neuropathy	115, 116, 117, 121, 124, 146, 147, 148
Carfilzomib	Kyprolis	Relapsed/refractory multiple myeloma, glioblastoma	High specificity; better curative effect for relapsed/refractory multiple myeloma; low incidence of peripheral neuropathy	High incidence of cardiac and renal toxicity	114, 125, 126, 127, 128
Ixazomib	Ninlaro	Relapsed/refractory multiple myeloma, neuroblastoma, hepatocellular carcinoma	Save medical costs; enrich relapsed/refractory multiple myeloma treatment methods; improve patients' quality of life	Single treatment plan; the curative effect advantage is not clear	114, 125, 129, 130, 132, 133
Marizomib	Salinosporamihe A	Multiple myeloma, glioblastoma	Not mention	Not mention	174, 175
Oprozomib	ONX-0912	Head and neck squamous cell carcinoma, hepatocellular carcinoma	Not mention	Not mention	178, 179
PR957	ONX-0914	Colitis-associated cancer, glioblastoma, lymphoblastic leukemia, prostate cancer	Not mention	Not mention	
DPLG3	Not mention	Immune diseases	Not mention	Not mention	
LU-005i	Not mention	Immune diseases	Not mention	Not mention	

## Generation I proteasome inhibitor: Bortezomib

Bortezomib is the first proteasome inhibitor as a chemotherapy drug,^172^^,^^173^ it can effectively bind to the β5 subunit of proteasome 20S and inhibit its catalytic activity for substrate degradation.^174^ MM can lead to an increase in serum proteasome levels, and successful chemotherapy can effectively restore the proteasome level to the normal range.^175^ Animal studies have shown that bortezomib may also have significant clinical effects on pancreatic cancer with extremely high mortality.^176^^,^^177^ Because bortezomib can act on both new diagnosis MM and relapsed/refractory MM, it has a wide range of indications.^173^ In addition, it can be combined with alkylating agents, immunomodulators, and monoclonal antibodies to form various schemes, so it has a wide range of clinical applications. Also, it has been reported that bortezomib can induce increased transcription and post-translation of cyclin-dependent kinase p21 and p27 in hepatocellular carcinoma, non-small cell lung cancer, breast cancer,^178^^,^^179^ and pancreatic cancer,^180^ which leads to growth inhibition and apoptosis in those tumor cells.^117^^,^^181^ Bortezomib, which has the ability to inhibit NF-κB signaling pathways and thus affect tumor occurrence, has also been reported in a variety of cancers including renal cancer, head and neck squamous cell carcinomas, and ovarian cancer.^124^, ^125^, ^126^

However, bortezomib has poor specificity; it inhibits β subunits while affecting other subunits, so it has certain damage to normal cells.^182^ At the same time, bortezomib is still resistant to some tumor patients in clinical application and may induce peripheral neuropathy.^173^ Therefore, the second-generation proteasome inhibitor carfilzomib was developed.

## Generation II proteasome inhibitor: Carfilzomib

In specificity, carfilzomib mainly acts on proteasome β5 subunit, and its ability to bind to other subunits is lower than bortezomib,^183^ so its specificity is better.^184^ Compared with bortezomib, carfilzomib has better curative effect on relapsed/refractory MM, and the incidence of peripheral neuropathy is lower.^172^ However, despite its high selectivity and irreversible advantages, its associated heart and kidney problems have raised clinical concerns.^185^^,^^186^ A recent study shows that carfilzomib can specifically induce tumor cell death and inhibit tumor growth when drug screening is carried out in glioblastoma, so protease inhibitors can be used as a potential targeted therapy for glioblastoma.^187^

## The first oral proteasome inhibitor: Ixazomib

Ixazomib is the first oral proteasome inhibitor.^183^^,^^188^ At present, ixazomib as a first-line treatment, is recommended in domestic and foreign countries to treat relapsed/refractory MM. The advantages of ixazomib are lower incidence of peripheral neuropathy, higher safety of heart and kidney than carfilzomib, and oral administration; these will save medical resources, improve patients' quality of life, and bring more benefits to relapsed/refractory MM patients.^172^^,^^189^ In addition, recent studies have demonstrated that ixazomib can induce apoptosis in neuroblastoma *in vitro* and *in vivo* by inhibiting doxorubicin-induced NF-κB activity and sensitizing neuroblastoma cells to doxorubicin-induced apoptosis.^190^ However, its disadvantage is that the treatment plan is single, and the curative effect advantage compared with other proteasome inhibitors is unknown.^191^^,^^192^ Sorafenib has been the standard treatment for hepatocellular carcinoma.^193^^,^^194^ However, recent studies have shown that the combination of ixazomib and the CDK inhibitor dinaciclib is effective in treating hepatocellular carcinoma. *In vitro* and *in vivo* studies have shown that ixazomib plus dinaciclib has synergistic pro-apoptotic and anti-proliferative activities and is superior to sorafenib in reducing patient-derived xenografts in mice.^195^

## Other proteasome inhibitors

There are other compounds currently in clinical trials, including marizomib, oprozomib, and delanzomib.^172^^,^^196^, ^197^, ^198^ Studies have found that marizomib (Salinosporamihe A) can not only treat MM but also cross the blood–brain barrier, so it can be used to treat glioblastomas.^199^^,^^200^ In addition, marizomib has promising anticancer activity in triple-negative breast cancer xenografts and can reduce lung and brain metastases by reducing the number of circulating tumor cells and the expression of genes involved in epithelial–mesenchymal transition.^201^ Oprozomib (ONX0912) is a truncated derivate of carfilzomib with better oral bioavailability compared with intravenously administrated carfilzomib.^202^ In head and neck squamous cell carcinoma xenograft models, carfilzomib and oprozomib were found to induce apoptosis in head and neck squamous cell carcinoma cells by up-regulating pro-apoptotic Bik.^203^ Oprozomib reduces the viability and proliferation of human hepatocellular carcinoma cells in a dose-dependent manner, it also has a good anti-tumor effect in xenograft hepatocellular carcinoma model.^204^

PR957 (ONX0914), as a proteasome inhibitor, specifically targets immunoproteasome β5i subunit and has been verified in several experienced models such as colitis-associated cancer, glioblastoma, acute lymphoblastic leukemia, and prostate cancer.^133^^,^^205^, ^206^, ^207^, ^208^, ^209^, ^210^, ^211^, ^212^ Moreover, DPLG3, a non-covalent β5i-specific inhibitor, and LU-005i, a pan-immunoproteasome inhibitor that targets all three active subunits, have shown therapeutic efficacy in immune diseases in mice.^210^^,^^213^ These results demonstrated that specific immunoproteasome inhibitors have a potential value to entrain a long-term response favorable to tumor suppression, and selectively affect the function of activated immune cells while sparing other cell types that would be damaged by treatment with classical proteasome inhibitors.

In the past 20 years, the treatment strategy of MM has changed greatly due to the introduction of proteasome inhibitors, which have significantly improved the survival and prognosis of patients. However, the side effects and curative effects of different proteasome inhibitors are not completely clear. Therefore, we need to know the characteristics of each proteasome inhibitor in further clinical trials and make the best treatment choice according to the specific condition of patients, to reduce the occurrence of adverse reactions. In addition, proteasome inhibitors have a variety of selectivity in the treatment of MM at present, but no significant therapeutic advantage has been found in other cancers. Therefore, it is an urgent problem to explore the role of proteasome inhibitors in other tumors, which may provide a new strategy for targeted tumor therapy.

## Conclusions and future directions

The process of life activities requires the metabolism of cells to maintain homeostasis, and the normal degradation of cycle-regulated proteins can make the cell cycle enter the normal order. Once the ability of cells to enter the S phase decreases, it will eventually lead to cell death or abnormal differentiation, and the abnormal DNA synthesis in cells will lead to the occurrence of tumors.^214^

Proteasome inhibitors have achieved great success in gaining clinical approval, which provides new directions for treatment strategies in different cancers. At present, proteasome inhibitors have been used as a first-line therapy in hematological tumors, but no significant advantage has been found in other tumors. However, the pro-apoptotic, anti-proliferative, and cytotoxic effects of bortezomib, as well as its negligible toxicity to normal hepatocytes, make it a potential new therapeutic approach for cancers. More and more studies have shown that by understanding the mechanism and driving factors of proteasomes in tumors, we can better combine proteasomes with other anticancer drugs to make excellent use of their advantages in treatment.^215^^,^^216^ For example, it can be used in combination with sorafenib to further down-regulate p-AKT and overcome apoptotic resistance in bortezomib resistance.^217^^,^^218^ Together with other kinase inhibitors such as chloroquine and mTOR inhibitors (rapamycin), they enhance apoptosis and reduce xenograft tumor size *in vitro*.^219^, ^220^, ^221^, ^222^ Similarly, bortezomib can function effectively in combination with gene drugs and histone deacetylase inhibitors.^223^, ^224^, ^225^, ^226^ Bortezomib can inhibit the development of tumors in different ways, not only through the classical pro-apoptotic pathway but also inhibit the metastasis of hepatocellular carcinoma via epithelial–mesenchymal transition process and cancer stem cells.^114^^,^^227^, ^228^, ^229^, ^230^

In conclusion, on the one hand, proteasome inhibitors have been developed as a therapeutic strategy for cancer treatment. These inhibitors can block the degradation of tumor suppressor proteins, leading to cell cycle arrest and apoptosis in cancer cells. On the other hand, proteasome inhibitors have been shown to have therapeutic potential in the treatment of various types of cancer, including MM, lymphoma, and solid tumors.

However, proteasome inhibitors can also have toxic effects on normal cells, and their use in cancer treatment must be carefully monitored. In addition, cancer cells can develop resistance to proteasome inhibitors, which can limit their effectiveness as a therapeutic strategy. Therefore, continued research is needed to better understand the role of proteasomes in cancer and to develop more effective and targeted therapies for cancer treatment.

## Conflict of interests

No potential conflict of interests was disclosed.

## Funding

This study was supported by the 10.13039/501100001809National Natural Science Foundation of China (No. 82172619), the 10.13039/501100005230Natural Science Foundation of Chongqing, China (No. CSTC2021jscx-gksb-N0023), and the Medical and Industrial Integration Project (China) (No. 2022CDJYGRH-002).
